# Supplementary material for: Analysis of Carica papaya Informs Lineage-Specific Evolution of the Aquaporin (AQP) Family in Brassicales
Source: Plants (Basel). 2023 Nov 3;12(22):3847. doi: 10.3390/plants12223847 (PMC10674200; doi:10.3390/plants12223847)
Supplement: Supplementary file 1 [file plants-12-03847-s001.zip › Figure S1.pdf]

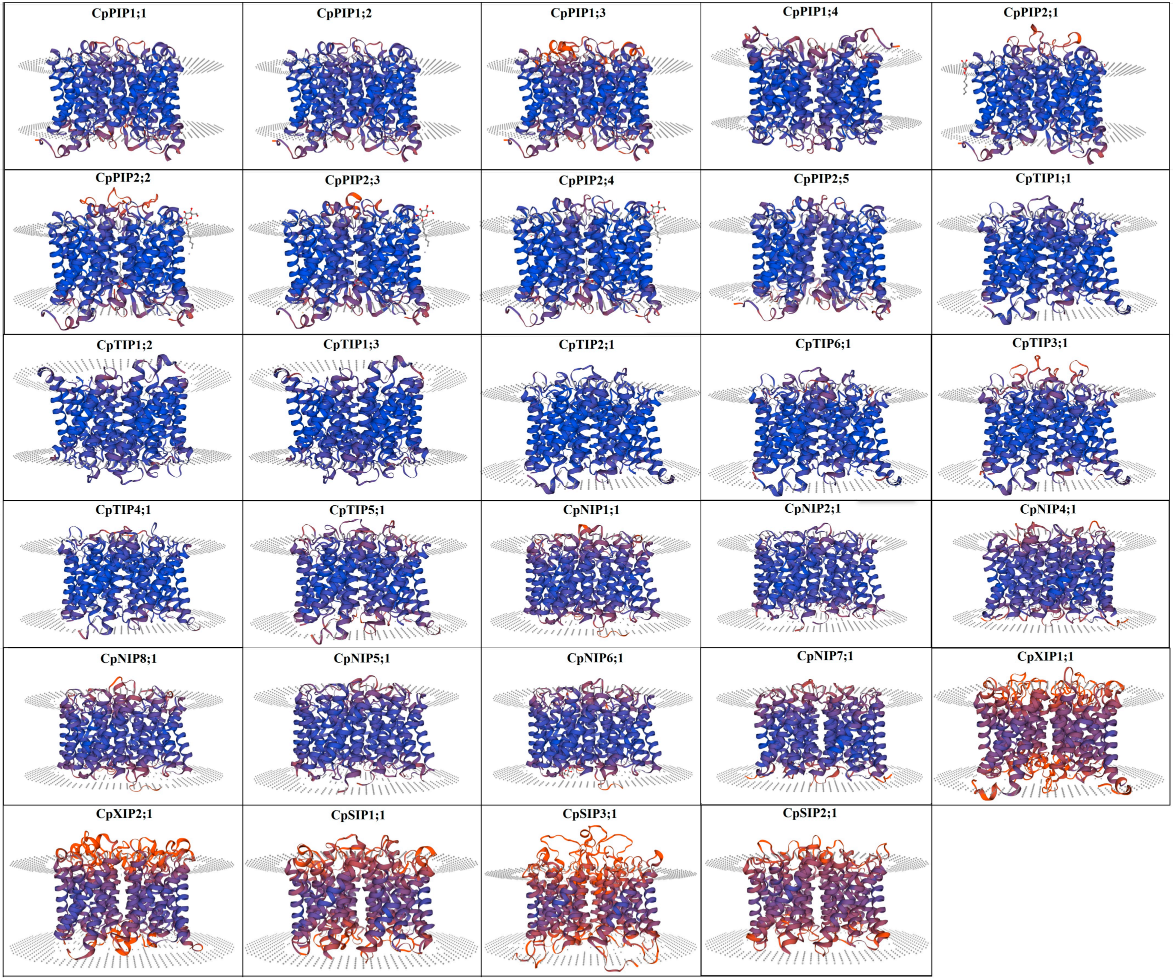
CpPIP1;1
CpPIP1;2
CpPIP1;3
CpPIP1;4
CpPIP2;1
CpPIP2;2
CpPIP2;3
CpPIP2;4
CpPIP2;5
CpTIP1;1
CpTIP1;2
CpTIP1;3
CpTIP2;1
CpTIP6;1
CpTIP3;1
CpTIP4;1
CpTIP5;1
CpNIP1;1
CpNIP2;1
CpNIP4;1
CpNIP8;1
CpNIP5;1
CpNIP6;1
CpNIP7;1
CpXIP1;1
CpXIP2;1
CpSIP1;1
CpSIP3;1
CpSIP2;1
