# Supplementary material for: Analysis of Carica papaya Informs Lineage-Specific Evolution of the Aquaporin (AQP) Family in Brassicales
Source: Plants (Basel). 2023 Nov 3;12(22):3847. doi: 10.3390/plants12223847 (PMC10674200; doi:10.3390/plants12223847)
Supplement: Supplementary file 1 [file plants-12-03847-s001.zip › File S1.pdf]

File S1 Alignment of CpAQPs with structure determined SoPIP2;1, AtTIP2;1, and OsNIP2;1. Multiple sequence alignment was performed using MUSCLE. Transmembrane helices (TM1–TM6) and the two short helices forming the two NPAs (HB and HE) (shaded), NPA motifs (shown in red), and ar/R selectivity filter residues (shown in green) are indicated. The highly conserved cysteine residues in XIPs are shown in **bold**. The residues at the position corresponding to S^{115} and S^{274} in SoPIP2;1（PDB accession number 1Z98）are highlighted in bright green, whereas the residues at the position corresponding to L^{197} from SoPIP2;1, determined to be the key residue involved in gating (occurring just before TM5) are underlined. The residues at the position corresponding to H^{131} in AtTIP2;1 (PDB accession number 5I32) are shown in yellow. The residues at the position corresponding to T^{109} in OsNIP2;1 (PDB accession number 7CJS) are shown in pink. The residues at the position corresponding to S^{262} in GmNOD26 (Genbank accession number P08995) are shown in dark green. "*", ":", and "." indicate consensus, strongly similar, and weakly similar amino acids, respectively.

```
SoPIP2;1        --------------MS--KEVSEEAQAHQHGKDYVDPPPAPFFDLGELKL----------
AtTIP2;1        -----------------------------------MAGVAFGS-FDDSFS----------
OsNIP2;1        ---------MASNNSRTNSRANYSNEIHDLSTVQNGTMPTMYYGEKAIAD----------
CpPIP1;1        MEGKEEDVKLGANKFSERQPIGTAAQTD---KDYKEPPPAPLFEPGELKS----------
CpPIP1;2        MEGKEEDVKLGANKFSERQPIGTSAQTD---KDYKEPPPAPLFEPGELHS----------
CpPIP1;3        MEGKEEDVRLGANKYSERQPIGTAAQSQDGGKDYKEPPPAPLFEPGELTS----------
CpPIP1;4        MEGKEEDVRLGANRYRERQPIGTAAQTQD-AKDYTEPPPAPLIEPGELFS----------
CpPIP2;1        --------------MA--KDIE--VGGEFQAKDYHDPPPAPLIDPEELTK----------
CpPIP2;2        --------------MT--KDVEVAEQGEFSAKDYHDPPPAPLIDVEELTK----------
CpPIP2;3        --------------MT--KDAG--EHGSFSAKDYHDPPPAPLFDAVELTK----------
CpPIP2;4        --------------MS--KEVSEEGQS--HGKDYVDPPPAPLIDLAEIKL----------
CpPIP2;5        --------------MSGGEGNEQTVGAEIRGRDYEDPPPASLIDMEELKK----------
CpTIP1;1        ---------------------------------MPIRNIAIGR-PEEATH----------
CpTIP1;2        ---------------------------------MPISQIAIGS-PAEIAR----------
CpTIP1;3        ---------------------------------MPIYRVAIGA-PRELSH----------
CpTIP2;1        -----------------------------------MACIAFGR-FDDIYS----------
CpTIP6;1        -----------------------------------MVKIALGS-FGDSFS----------
CpTIP3;1        ---------------------------------MPVRRYAFGR-VEEVTH----------
CpTIP4;1        -----------------------------------MAKIALGT-RHEVTK----------
CpTIP5;1        -----------------------------------MAPASLTSRFGQSIT----------
CpNIP1;1        -------MAEISMNSANNNRVVLNVNDGDVGHNSLPASTSESLEKRDSAL----------
CpNIP2;1        ---------MAG--TLTHPNLNNQTDINDLVSVES-PISDRSSIWKSFEH----------
CpNIP4;1        --------------MASKNDSIEEVPPDVEEEGTATSTNRLEPTSKNSSN----------
CpNIP8;1        --------------MSHQDYEVSKVEEGEA------CSRSSSRIPESSST----------
CpNIP5;1        -----MPESETGTPTASAPATPGTPGGPLISGLRVDSLS---YDRKSMAR-CKCLPVTAP
CpNIP6;1        -----MDQKEDVPSAPSTPATPGTPGAPLFGGFRRERSSSGFFRSKSLINTCNCFTLRDW
CpNIP7;1        --------------MKIKNFLNPQSSPDDQNTNGFINTTIFPLHCLSLQM----------
CpXIP1;1        -----MESVIVSSDRSTHSAL------STSIENYDKTSPTRISKNNSFLASIG-------
CpXIP2;1        -MARNGGAIVVEDEENPYSATRIQPVASTPMAQQRNTEKGKKKTPTTLTGVLG-------
CpSIP1;1        ------------------------------------------------------------
CpSIP3;1        ------------------------------------------------------------
CpSIP2;1        ------------------------------------------------------------
```

TM1

```
SoPIP2;1    -------------------WSFWRAAIAEFIATLLFLYITVATVIGHSKETV-------V
AtTIP2;1    -------------------LASLRAYLAEFISTLLFVFAGVGSAIAYAKLTSDA-----A
OsNIP2;1    ----------------FFPPHLLKKVVSEVVATFLLVFMTCGAAGISGSDLS--------
CpPIP1;1    -------------------WSFYRAGIAEFIATFLFLYITILTVMGVKRSDS-------M
CpPIP1;2    -------------------WSFWRAGIAEFMATFLFLYITVLTVMGVNRAPN-------K
CpPIP1;3    -------------------WSFYRAGIAEFMATFLFLYITVLTVMGVVKESS-------K
CpPIP1;4    -------------------WSFYRAGIAEFVATFLFLYITVLTVMGVVKSPT-------K
CpPIP2;1    -------------------WSFYRALTAEFVATLLFLYITVLTVIGYKSQTD-KNQGGDD
CpPIP2;2    -------------------WSFYRALIAEFVATLLFLYVTVLTVIGHKSQTDLTKAGTDV
CpPIP2;3    -------------------WSFYRALIAEFIATLLFLYVTVLTVIGHKSQH-----AADQ
CpPIP2;4    -------------------WSFYRALIAEFIATLLFLYVTIATVIGHKKQAG-------P
CpPIP2;5    -------------------WSFYRAVIAEFVATFLFLYVGIATVIGNKKQIH-------P
CpTIP1;1    -------------------PDALKAALAEFISMLIFVFAGEGSGMAFNKLTNNG-----A
CpTIP1;2    -------------------PDALKAALAEFISMLIFVFAGEGSGMAFNKLTNYG-----S
CpTIP1;3    -------------------PSAIRAALAEFFSMVIFVFAGEGSGMAFDKLTNNG-----S
CpTIP2;1    -------------------LASLKAYLAEFISTLLFVFAGVGSAMAFNKLTSDA-----P
CpTIP6;1    -------------------VGSLKAYLSEFIATLLFVFAGVGSAIAFGKVTSDG-----A
CpTIP3;1    -------------------PDSIRATLAEFLSTLVFVFAGEGSVLALDKLYRETG-GDVS
CpTIP4;1    -------------------PDCIRALVVEFITTFLFVFTGVGSAMAADRLVGNG------
CpTIP5;1    -------------------KNAFRSYLAEFISTFFYVLTVVGSSMSARKLMGSG-----A
CpNIP1;1    ----------------SFSVPFIQKLMAEFFGTFFLIFAGCASVAVNDEYEK--------
CpNIP2;1    ----------------HYPPCFLRKVAAEVIATYLLVFVTCGSAAISAIDES--------
CpNIP4;1    ----------------LWAFTLAQKVIAELVGTYFIIFSGCGAVAVNKIYG---------
CpNIP8;1    ----------------SWVVELLQKVIAEVLGTYFVMFSGCGSVVVNKIYG---------
CpNIP5;1    TWGQP---HTCFIDFPAPDVSLTRKLGAEFVGTFILIFAATAAPIVNQKYNG--------
CpNIP6;1    SLEDPSVLPPVTCTLPHPPVSLARKVGAEFIGTLILIFAGTATAIVNQKSQG--------
CpNIP7;1    ------------------DFNPARAILAEMVGTFILVLCVCGIIASQQLMRG--------
CpXIP1;1    -------------AHEFFSQEMWGAAITELVGTTCLLFTLTISIVACLNFHE--------
CpXIP2;1    -------------FKDLFSLKVWRASLAELLGTAVLVFAMDTIVISSYETQT--------
CpSIP1;1    -------------------MGPIKAAIGDMILTFMWNFFASTFGLMT--VMILGAAGLQG
CpSIP3;1    -------------------MGVIRSAIGDSVLTSMWVFNLPVIGLLAGRASDFLRTHYIS
CpSIP2;1    -------------------MAKIRLLISDFIVSFMWVWSGVLIKIFVHRVLGLGHEPRAE
                                              :  .
```

```
                        TM2
SoPIP2;1        CGSVGLLGIAWAFGGMIFVLVYCTAGISGGHINPAVTFGLFLARKVSLLRALVYMIAQCL
AtTIP2;1        LDTPGLVAIAVCHGFALFVAVAIGANISGGHVNPAVTFGLAVGGQITVITGVFYWIAQLL
OsNIP2;1        --RISQLGQSIAGGLIVTVMIYAVGHISGAHMNPAVTLAFAVFRHFPWIQVPFYWAAQFT
CpPIP1;1        CASVGIQGIAWAFGGMIFALVYCTAGISGGHINPAVTFGLLLARKLSLTRAVFYMIMQCL
CpPIP1;2        CASVGIQGIAWAFGGMIFALVYCTAGISGGHINPAVTFGLFLARKLSLTRSIFYMVMQCL
CpPIP1;3        CSTVGIQGIAWAFGGMIFALVYCTAGISGGHINPAVTFGLLLARKLSLTRAIFYMVMQCL
CpPIP1;4        CSTVGIQGIAWAFGGMIFALVYCTAGISGGHINPAVTFGLLLARKLSLVRAVFYMIMQCL
CpPIP2;1        RGGVGILGIAWAFGGMIFILVYCTAGISGGHINPAVTFGLFLARKVSLVRAVLYMVAQCL
CpPIP2;2        CGGVGLLGIAWAFGGMIFILVYCTAGISGGHINPAVTLGLFLARKVSLIRAIMYMVAQCL
CpPIP2;3        CGGVGILGIAWAFGGMIFILVYCTAGISGGHINPAVTFGLFLARKVSLVRAVMYMVAQCL
CpPIP2;4        CDGVGILGIAWAFGGMIFILVYCTAGISGGHINPAVTFGLFLARKVSLIRAVAYMVAQCL
CpPIP2;5        CDGVGLLGISWSFGGMIFILVYCTAGISGGHINPAVTLGLFAARKMSLIRAAAYMVSQCG
CpTIP1;1        ATPAGLIAAAIAHAFALFVAVAVGANISGGHVNPAVTFGAFVGGNISLLRGILYWIAQLL
CpTIP1;2        ATPAGLISASLAHAFALFVAVSVGANISGGHVNPAVTFGAFVGGHISLFRSVLYWIAQCL
CpTIP1;3        STPAGLVAASLAHAFALFVAVSVGANISGGHVNPAVTFGAFLGGNITFFRSILYWIAQLL
CpTIP2;1        LDPSGLVAIAVCHGFALFVAVAVAANISGGHVNPAVTLGLAVGGQITILTGIFYWIAQLL
CpTIP6;1        LDPAGLVAIAVAHAFALFVGVAIAANISGGHLNPAVTLGLAIGGNITLLTGLFYWIAQCL
CpTIP3;1        RDPSGLVLIALAHALSLFSAVSASINISGGHVNPAVTFGALLGGRISVLRAFYYWLAQLL
CpTIP4;1        --LLGLFAVAVAHALVVAVMISAG-HISGGHLNPAVTLGLLFGGHITLVRSILYWIDQLL
CpTIP5;1        EDPSGLVIVAIANSLGLSYTVYVASNISGGHVNPAVTFARAVGGHVSVPTALFYWVSQML
CpNIP1;1        --VVTLPGISIVWGLAVMVLVYSVGHISGAHFNPAVTIAFASCRRFPLKQVPAYILVQLL
CpNIP2;1        --RVSKLAASVAGGLIVTVMIYAVGHISGAHMNPAVTLAFAALRHFPWKQVPFYAAAQVT
CpNIP4;1        --SVTFPGICVTWGLIVTVMIYTVGHVSGAHFNPAVTIAFTIFQKFPPSEVLFYIVAQFL
CpNIP8;1        --SVTFPGICVVWGLIVMVMVYSVGHISGAHFNPAVTITFAIFRRFPFKQVPLYILAQLL
CpNIP5;1        --AETLIGNAACSGLAVMIIILSTGHISGAHLNPSVTICFAALRHFPWAQVPAYIAAQIS
CpNIP6;1        --SETLIGLAASTGLAVMIVILSTGHISGAHLNPAVTISFAALHHFPWKHVPAYIAAQTV
CpNIP7;1        --EVGLMEYAATAGLTVVVVIFCIGPISGAHVNPAVTIAFAIFGHFSWSRVPFYILAQML
CpXIP1;1        --VESKLLVPIVVFVILFFFLMATIPISGGHMNPVFTFIATLKGIITITRAAFYFLAQCL
CpXIP2;1        --KTPHLIMSFLVAITVTILLLATSPISGGHINPIVTVAAVLTGLISVSRAIVYILAQCI
CpSIP1;1        VAWAPVLITTLLVFIFVFIFGLIGDALGGASFNPTGTAAFYAAGVG--SDSLLSMAIRFP
CpSIP3;1        LPFTGLFITILLATINVLLFTLLGTLLGGASFNPSTTVSFHAAGLTKPGSSLISMAVRLP
CpSIP2;1        IIRG------AMAIVNMFFFAFLGKVGKGASYNPLTVLAPAVSGDF--SSFLFSLGCRIP
                                :           *.  **  .                    :
```

```
                                                  HB               TM3
SoPIP2;1      CGSVGLLGIAWAFGGMIFVLVYCTAGISGGHINPAVTFGLFLARKVSLLRALVYMIAQCL
AtTIP2;1      LDTPGLVAIAVCHGFALFVAVAIGANISGGHVNPAVTFGLAVGGQITVITGVFYWIAQLL
OsNIP2;1      --RISQLGQSIAGGLIVTVMIYAVGHISGAHMNPAVTLAFAVFRHFPWIQVPFYWAAQFT
CpPIP1;1      CASVGIQGIAWAFGGMIFALVYCTAGISGGHINPAVTFGLLLARKLSLTRAVFYMIMQCL
CpPIP1;2      CASVGIQGIAWAFGGMIFALVYCTAGISGGHINPAVTFGLFLARKLSLTRSIFYMVMQCL
CpPIP1;3      CSTVGIQGIAWAFGGMIFALVYCTAGISGGHINPAVTFGLLLARKLSLTRAIFYMVMQCL
CpPIP1;4      CSTVGIQGIAWAFGGMIFALVYCTAGISGGHINPAVTFGLLLARKLSLVRAVFYMIMQCL
CpPIP2;1      RGGVGILGIAWAFGGMIFILVYCTAGISGGHINPAVTFGLFLARKVSLVRAVLYMVAQCL
CpPIP2;2      CGGVGLLGIAWAFGGMIFILVYCTAGISGGHINPAVTLGLFLARKVSLIRAIMYMVAQCL
CpPIP2;3      CGGVGILGIAWAFGGMIFILVYCTAGISGGHINPAVTFGLFLARKVSLVRAVMYMVAQCL
CpPIP2;4      CDGVGILGIAWAFGGMIFILVYCTAGISGGHINPAVTFGLFLARKVSLIRAVAYMVAQCL
CpPIP2;5      CDGVGLLGISWSFGGMIFILVYCTAGISGGHINPAVTLGLFAARKMSLIRAAAYMVSQCG
CpTIP1;1      ATPAGLIAAAIAHAFALFVAVAVGANISGGHVNPAVTFGAFVGGNISLLRGILYWIAQLL
CpTIP1;2      ATPAGLISASLAHAFALFVAVSVGANISGGHVNPAVTFGAFVGGHISLFRSVLYWIAQCL
CpTIP1;3      STPAGLVAASLAHAFALFVAVSVGANISGGHVNPAVTFGAFLGGNITFFRSILYWIAQLL
CpTIP2;1      LDPSGLVAIAVCHGFALFVAVAVAANISGGHVNPAVTLGLAVGGQITILTGIFYWIAQLL
CpTIP6;1      LDPAGLVAIAVAHAFALFVGVAIAANISGGHLNPAVTLGLAIGGNITLLTGLFYWIAQCL
CpTIP3;1      RDPSGLVLIALAHALSLFSAVSASINISGGHVNPAVTFGALLGGRISVLRAFYYWLAQLL
CpTIP4;1      --LLGLFAVAVAHALVVAVMISAG-HISGGHLNPAVTLGLLFGGHITLVRSILYWIDQLL
CpTIP5;1      EDPSGLVIVAIANSLGLSYTVYVASNISGGHVNPAVTFARAVGGHVSVPTALFYWVSQML
CpNIP1;1      --VVTLPGISIVWGLAVMVLVYSVGHISGAHFNPAVTIAFASCRRFPLKQVPAYILVQLL
CpNIP2;1      --RVSKLAASVAGGLIVTVMIYAVGHISGAHMNPAVTLAFAALRHFPWKQVPFYAAAQVT
CpNIP4;1      --SVTFPGICVTWGLIVTVMIYTVGHVSGAHFNPAVTIAFTIFQKFPPSEVLFYIVAQFL
CpNIP8;1      --SVTFPGICVVWGLIVMVMVYSVGHISGAHFNPAVTITFAIFRRFPFKQVPLYILAQLL
CpNIP5;1      --AETLIGNAACSGLAVMIIILSTGHISGAHLNPSVTICFAALRHFPWAQVPAYIAAQIS
CpNIP6;1      --SETLIGLAASTGLAVMIVILSTGHISGAHLNPAVTISFAALHHFPWKHVPAYIAAQTV
CpNIP7;1      --EVGLMEYAATAGLTVVVVIFCIGPISGAHVNPAVTIAFAIFGHFSWSRVPFYILAQML
CpXIP1;1      --VESKLLVPIVVFVILFFFLMATIPISGGHMNPVFTFIATLKGIITITRAAFYFLAQCL
CpXIP2;1      --KTPHLIMSFLVAITVTILLLATSPISGGHINPIVTVAAVLTGLISVSRAIVYILAQCI
CpSIP1;1      VAWAPVLITTLLVFIFVFIFGLIGDALGGASFNPTGTAAFYAAGVG--SDSLLSMAIRFP
CpSIP3;1      LPFTGLFITILLATINVLLFTLLGTLLGGASFNPSTTVSFHAAGLTKPGSSLISMAVRLP
CpSIP2;1      IIRG------AMAIVNMFFFAFLGKVGKGASYNPLTVLAPAVSGDF--SSFLFSLGCRIP
                              :           *.  **  .                    :
```

```
                                                               TM4
SoPIP2;1        GAICGVGLVKAFM-KGPYNQFGGG-----------ANSVALGYNKGTALGAEIIGTFVLV
AtTIP2;1        GSTAACFLLKYVTGGLAVPTHS----------------VAAGLGSIEGVVMEIIITFALV
OsNIP2;1        GAICASFVLKAVIHP-VDVIGT--------------TTPVGPHW--HSLVVEVIVTFNMM
CpPIP1;1        GAICGAGVVKGFQ-PGPYQRLGGG-----------ANVVNHGYTKGDGLGAEIVGTFVLV
CpPIP1;2        GAICGAGVVKGFQ-PRPYQMLGGG-----------ANMVNHGYTKGDGLGAEIVGTFVLV
CpPIP1;3        GAICGAGVVKGFEGSATFELKGGG-----------ANVVNHGYTKGDGLGAEIVGTFVLV
CpPIP1;4        GAICGAGVVKAFE-KTQYEMLGGG-----------ANTVGPAYSKTAGLGAEIVGTFVLV
CpPIP2;1        GAICGCGLVKAFQ-KACYNRYGGG-----------ANQLALGYSTGTGLGAEIIGTFVLV
CpPIP2;2        GAICGVGLVKAFQ-SSFYNRYGGG-----------ANSLNGGYNKGTGLGAEIIGTFVLV
CpPIP2;3        GAICGVGLVKAFQ-KSFYNRFGGG-----------ANTLADGYNVGTGLGAEIIGTFVLV
CpPIP2;4        GAICGVGLVKAFM-KNYYNRLGGG-----------ANTVATGYNTGTALGAEIIGTFVLV
CpPIP2;5        GAICGVGLVKLFM-TRSYNMHGGG-----------ANSVAPGFSTTTGLGAEIIGSFVLV
CpTIP1;1        GSVAACALLKFATGGLTTSAFA----------------LSSGVGVWNAFVFEIVMTFGLV
CpTIP1;2        GSVLACLLLKFSTGGLETSAFA----------------LSSGVGELNALVFEIVMTFGLV
CpTIP1;3        GSVVACLLLKFATGNMETAAFG----------------LSSGVSPMNALVFEIVMTFGLV
CpTIP2;1        GSIVACFLLKAVTGCMAIPIHS----------------VAAEVGVIGGLVMEIIITFALV
CpTIP6;1        GSIVACGLLKFVT-DLSVPTHS----------------VGSGMSVLEGVVMEIVITFALV
CpTIP3;1        GAIVACLLLRLVTAGMRPVGFR----------------VASGVGELNGLVLEMVLTFGLM
CpTIP4;1        ASSIACILLKYLTGGLDTPIHT----------------LASGVGYGQGVVWEIVLTFSLL
CpTIP5;1        ASVMASLILRVMTVAQHVPTYA----------------IAEQMTGFGASVLEGVLTFALV
CpNIP1;1        GSTLAAGTLRLLFNGQHQVFTG--------------TAPSGSDM--QSFGIEFIITFYLM
CpNIP2;1        GAISAAFTLRVLLHP-IKLIGT--------------TSPAGSDI--QALIMEIVVTFSMM
CpNIP4;1        GSILASGTLALMFDITPNAYFG--------------TTPVGSNG--QSLAIEIIITFLLT
CpNIP8;1        GAILASYTLWIIFHVNEESFFG--------------TVPVGSDL--QSLWIEIIISFILM
CpNIP5;1        ASICASFTLKGVFHP-FLSGGV--------------TVPSVSLG--QAFALEFLISFILL
CpNIP6;1        GSLCAAFALKIVFHP-MMGGGV--------------TVPSPSVGYAQAFALEFIISFNLM
CpNIP7;1        GSTLATWAGRSVYGVRADLMAT--------------RPVQGCFA---AFWVEFFGTFIIM
CpXIP1;1        GSIISFIIIKSVMNHDSATKFSLGGCSIKGHGS-------TGLHLGVALMLEFSCTFLVL
CpXIP2;1        GGILGALALKAVVNSTIQQTFSLGGCTLTVVVPGRHGPVVIGLETGQALWLEIICTFVFL
CpSIP1;1        AQAVGAVGGALAITEVMPIQYKHM-----------IGGPSLKVELHTGAVAEGILTFLIT
CpSIP3;1        AQAAGGAIGAMGIWQVMPVG--WL-----------KGGPSLKVDWHTGALAEGLLAFAHC
CpSIP2;1        VQVIGSIVGVRFILETIPEAG---------------LGPRLKVDIHQGALTEGFLTFAIV
                    .                                          .   *   :*
```

```
                                          TM5                     HE
SoPIP2;1    YTVFSATDPKRSARDS---HVPILAPLPIGFAVFMVHLATIP--ITGTGINPARSFGAAV
AtTIP2;1    YTVYATAADPKKG------SLGTIAPLAIGLIVGANILAAGP--FSGGSMNPARSFGPAV
OsNIP2;1    FVTLAVATDTRAVG--------ELAGLAVGSAVCITSIFAGA--ISGGSMNPARTLGPAL
CpPIP1;1    YTVFSATDAKRNARDS---HVPILAPLPIGFAVFLVHLATIP--ITGTGINPARSLGAAI
CpPIP1;2    YTVFSATDAKRNARDS---HVPILAPLPIGFAVFLVHLATIP--ITGTGINPARSLGAAI
CpPIP1;3    YTVLSATDAKRNARDS---HVPILAPLPIGFAVFLVHLATIP--ITGTGINPARSLGAAI
CpPIP1;4    YTVFSATDAKRNARDS---HVPILAPLPIGFAVFLVHLATIP--VTGTGINPARSLGAAL
CpPIP2;1    YTVFSATDPKRNARDS---HVPVLAPLPIGFAVFMVHLATIP--VTGTGINPARSFGAAV
CpPIP2;2    YTVFAATDPKRNARDS---HVPVWAPLPIGFAVFMVHLATIP--ITGTGINPARSFGAAV
CpPIP2;3    YTVFSATDPKRSARDS---HVPVLAPLPIGFAVFMVHLATIP--VTGTGINPARSFGAAV
CpPIP2;4    YTVFSATDPKRSARDS---HVPVLAPLPIGFAVFMVHLATIP--ITGTGINPARSFGAAV
CpPIP2;5    YTVFSATDPKRSARES---FIPVLAPLPIGLAVFMVHLATIP--ITGTGINPARSLGAAV
CpTIP1;1    YTVYATAVDPKKG------SLGTIAPIAIGFIVGANILAGGA--FDGASMNPAVSFGPAV
CpTIP1;2    YTVYATAIDPKRG------NIGIIAPIAIGFIVGANILAGGA--FDGASMNPAVSFGPAV
CpTIP1;3    YTVYATAVDPKKG------NLGTIAPIAIGFIVGANILAGGA--FDGASMNPAVSFGPAV
CpTIP2;1    YTVYATAADPRKG------SLGTIAPIAIGFIVGANILAAGP--FSGGSMNPARSFGPAV
CpTIP6;1    YTVYATAADPKRG------SLGIIAPIAIGFIVGANILAAGP--FSGGSMNPARSFGPAV
CpTIP3;1    YTVYATTIDPKRG------SIGIIGPLAIGLIVGGNILVGGP--FDGGSMNPARAFGPAL
CpTIP4;1    FTVYATIVDPKKG------SLDGLGPMLTGFVVGANILAGGA--FSGASMNPARSFGPAL
CpTIP5;1    YTIYAAG-DPRRG------QMGAIGPLVIGMAAGANFLAAGP--FSGGSMNPACAFGSAV
CpNIP1;1    FIISGVATDNRAIG--------ELAGLAVGATVLLNVMFAGP--ISGASMNPARSLGPAI
CpNIP2;1    FITSAVATDTKAVG--------ELAGIAVGSAVCITSILAGP--VSGGSMNPARSIGPAL
CpNIP4;1    FVIFGASIDERAIG--------QLGGIAVGMTVMLNVFVAGP--ISGASMNPARSLGPAF
CpNIP8;1    FVISGVATDNRAIG--------ELAGIAVGMTIILNVFVAGP--VSGASMNPARSIAPAI
CpNIP5;1    FVITAVATDTRAVG--------ELAGIAVGATVMLNILVAGP--SSGGSMNPVRTLGPAV
CpNIP6;1    FVVTAVATDTRAVG--------ELAGIAVGATVMLNILIAGP--STGASMNPVRTLGPAI
CpNIP7;1    FLSAALICEAHTIG--------HLSGFVVGIAIGLAVLITGP--VSGGSMNPARSLGPAI
CpXIP1;1    FVAVNVAFDKRRSKELGVSKVCAQIAGAMALAVFVSITVTGQTAYAGAGLNPAKCFGAAI
CpXIP2;1    FASIWVAFDYRQAKALGRFMVCLVIGVVVGLIVFVSTTVTATKGYAGVGMNPARCLGPAL
CpSIP1;1    FAVLVIILKGPRNS--------VLKTWLLAVATVALVLSGSA--YTGPAMNPAIAFGWAY
CpSIP3;1    LSVLVVVVRGPRSV--------FVKVLLLAMVTTGLVRVGSG--YTGPSLNPANAFGWAY
CpSIP2;1    MISLGLAAKIPGSF--------FMKTWISSVSKLALHILGSD--LTGGCMNPASVMGWAF
                                          .               *  :**.  :. *
```

TM6

```
SoPIP2;1    IFNSNKVWDDQWIFWVGPFIGAAVAAAYHQYVLRAAAI-------KALGSFRSNPTN---
AtTIP2;1    AAGD---FSGHWVYWVGPLIGGGLAGLIYGNVFMGSSE-------HVPLASADF------
OsNIP2;1    ASNK---FDGLWIYFLGPVMGTLSGAWTYTFIRFEDTPKEGSSQKLSSFKLRRLRSQQSI
CpPIP1;1    IFNTDHAWDDHWIFWVGPFIGAALAAVYHQIVIRAIPF-------KTRA-----------
CpPIP1;2    IYNDDTAWDHHWVFWVGPFIGAALAALYHQIVIRAIPF-------KTRG-----------
CpPIP1;3    IFNRDKAWDDHWIFWVGPFIGAALAALYQQVVIRAIPF-------KSK------------
CpPIP1;4    IYNKSQAWDDHWIFWVGPFIGAALAALYHQIVIRAIPF-------RSK------------
CpPIP2;1    IFNQDKPWDDHWIFWVGPFIGAAIAAFYHQFVLRASGS-------KSLGSLRSSSNI---
CpPIP2;2    IFNDEKAWDDHWIFWVGPFIGAAIAAIYHQYVLRAAAI-------KALGSFRSNA-----
CpPIP2;3    IFNDKKAWDDHWIFWVGPFIGAAIAAFYHQFILRAAAV-------KALGSFRSQSHV---
CpPIP2;4    IWNNKKGWDDHWIFWVGPFVGALAAAAYHQYILRAAAI-------KALGSFRSNPTN---
CpPIP2;5    VYNNQQVWDEQWIFWVGPFLGALAAAAYHEYVLRAAAV-------KALLSFRPH------
CpTIP1;1    VSWS---WDNHWVYWAGPLIGGGLAGLIYDFFFISHS--------HEQLPTADY------
CpTIP1;2    VSWT---WNSHWVYWVGPFAGAGIAALVYEIIFIGSST-------HEQLASADF------
CpTIP1;3    VSWT---WTHHWVYWVGPFIGAAIAAIVYDNIFIGNDS-------HEPLPTNDF------
CpTIP2;1    ASGN---FCGIWIYWVGPLVGGGLAGLIYGNIFISSS--------HQSLPSDC-------
CpTIP6;1    VSGD---FTDNWVYWLGPLVGGALAGLVYGDIFIGSYS-------PVSASQDYP------
CpTIP3;1    VGWR---WRNHWIYWVGPFVGGGLAALVYEFMVIPSTTEPPLITGHQPLAPEDY------
CpTIP4;1    VSWD---WTDHWVYWVGPLMGGGLAGFMYENFFIVRS--------HVLVSHQDDSC----
CpTIP5;1    VAGS---FKNQAVYWVGPLIGATIGGLLYDNVVFPPEAIDS----LTGISERPVV-----
CpNIP1;1    VFSR---YKGIWIYIFSPVLGAISGAWVYNMVRYTDKPLREITKSSSFLKSARSCST---
CpNIP2;1    ASQY---YKGIWVYLVGPVTGTLLGAYSYNLIRVKDEPVQAISPRSFSFKLRRMKS-HEE
CpNIP4;1    VKHE---FKGLWIYVIGPVAGAIAGASAYSLVRAGDRP--------SETLSFLTGSSK--
CpNIP8;1    VMHV---YKGLWVYIVGPPIGTILGGAAYNLIRFTDKPLREITRTGSFLKSISRRN----
CpNIP5;1    AAGN---YKAIWVYLLAPILGGLVGAGTYTAVKLRDDEAE---PPRQVRSFRR-------
CpNIP6;1    AANN---YKAIWVYLIAPILGALSGAGIYTAVKLPEEDADTHEKPSTARSFRR-------
CpNIP7;1    VSWD---FDNIWIYMIAPVVGAIAGVLLYQFLRLKHRPCTATSSPSTVSYLVTP------
CpXIP1;1    LQGG-LLWKGHWVFWVGSFFACIVYYGFSLTLPKQGLDWVEGEYDAMRLAKACWGTKDFP
CpXIP2;1    IRGG-HLWSGHWVFWAGPVIACVAFALYIKMIPREHFHGGD-------------------
CpSIP1;1    QNNWHNSWEHFYVYWICPFIGAIFAALVFRIIFPPTEVK------KKKQKKA--------
CpSIP3;1    VKNWHNSLELYYVYWVGPLVGATMAAWVFRVLFAPSLVK------KKKKKKKRE------
CpSIP2;1    ARGDHITKEHIFVYWLAPVEATLLAVWTFKLVTKSLTED------KAKLKAKSE------
                        ::   .  .          .
```
