# Supplementary figures and images for: Analysis of Carica papaya Informs Lineage-Specific Evolution of the Aquaporin (AQP) Family in Brassicales
Source: Plants (Basel). 2023 Nov 3;12(22):3847. doi: 10.3390/plants12223847 (PMC10674200; doi:10.3390/plants12223847)

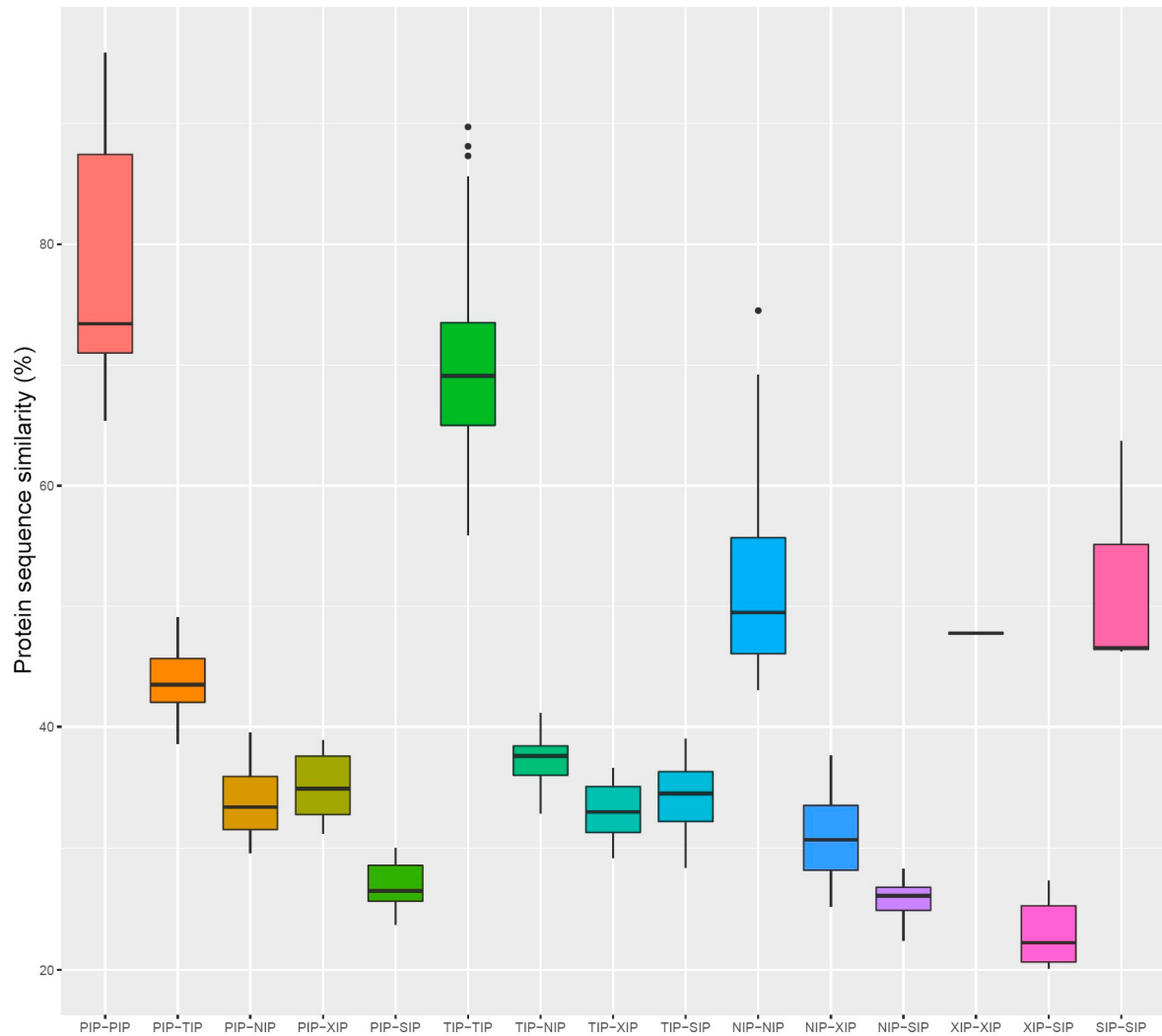

Supplement: Supplementary file 1 [file plants-12-03847-s001.zip › Figure S2.pdf]

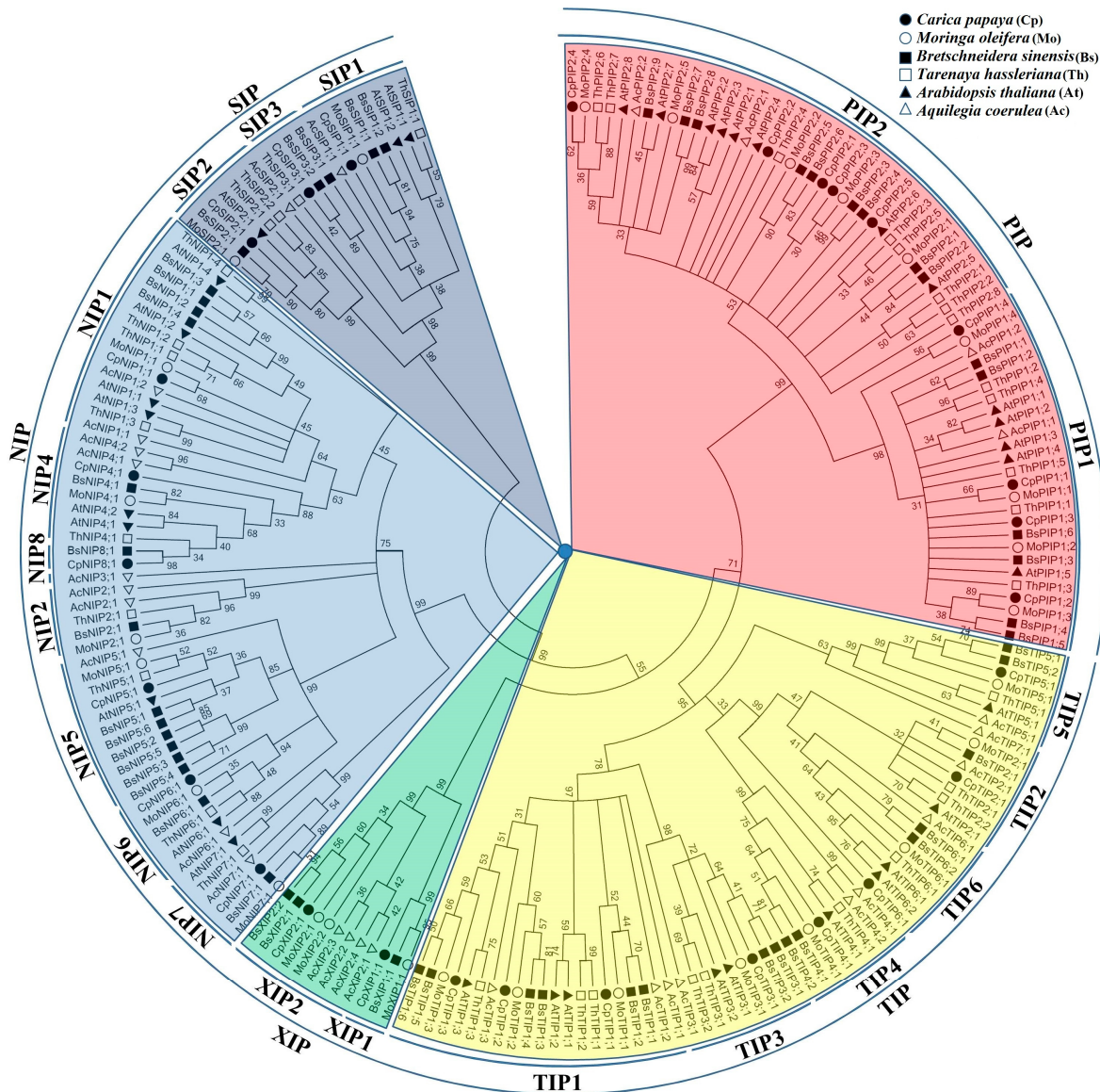

Supplement: Supplementary file 1 [file plants-12-03847-s001.zip › Figure S3.pdf]

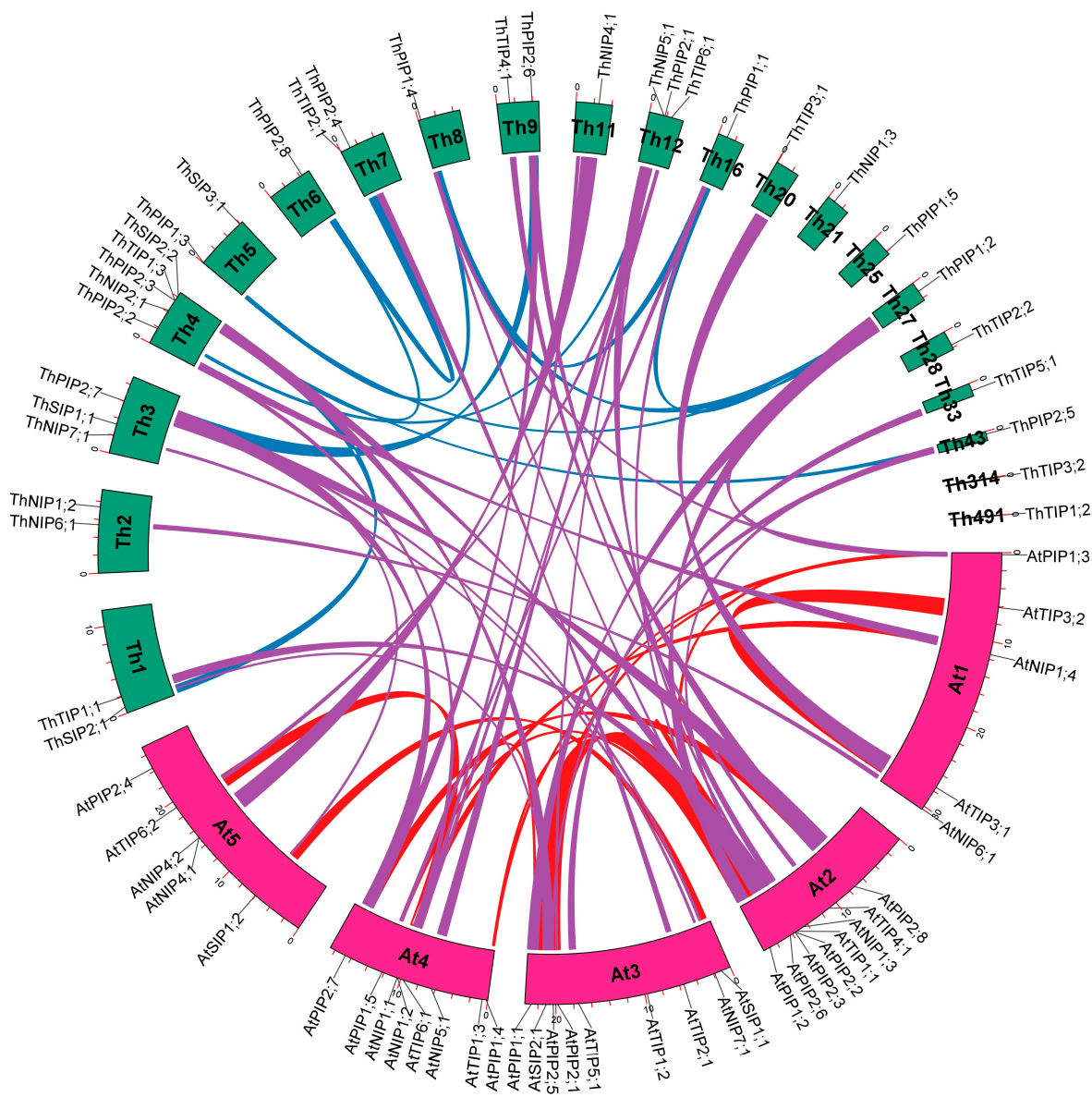

Supplement: Supplementary file 1 [file plants-12-03847-s001.zip › Figure S4.pdf]

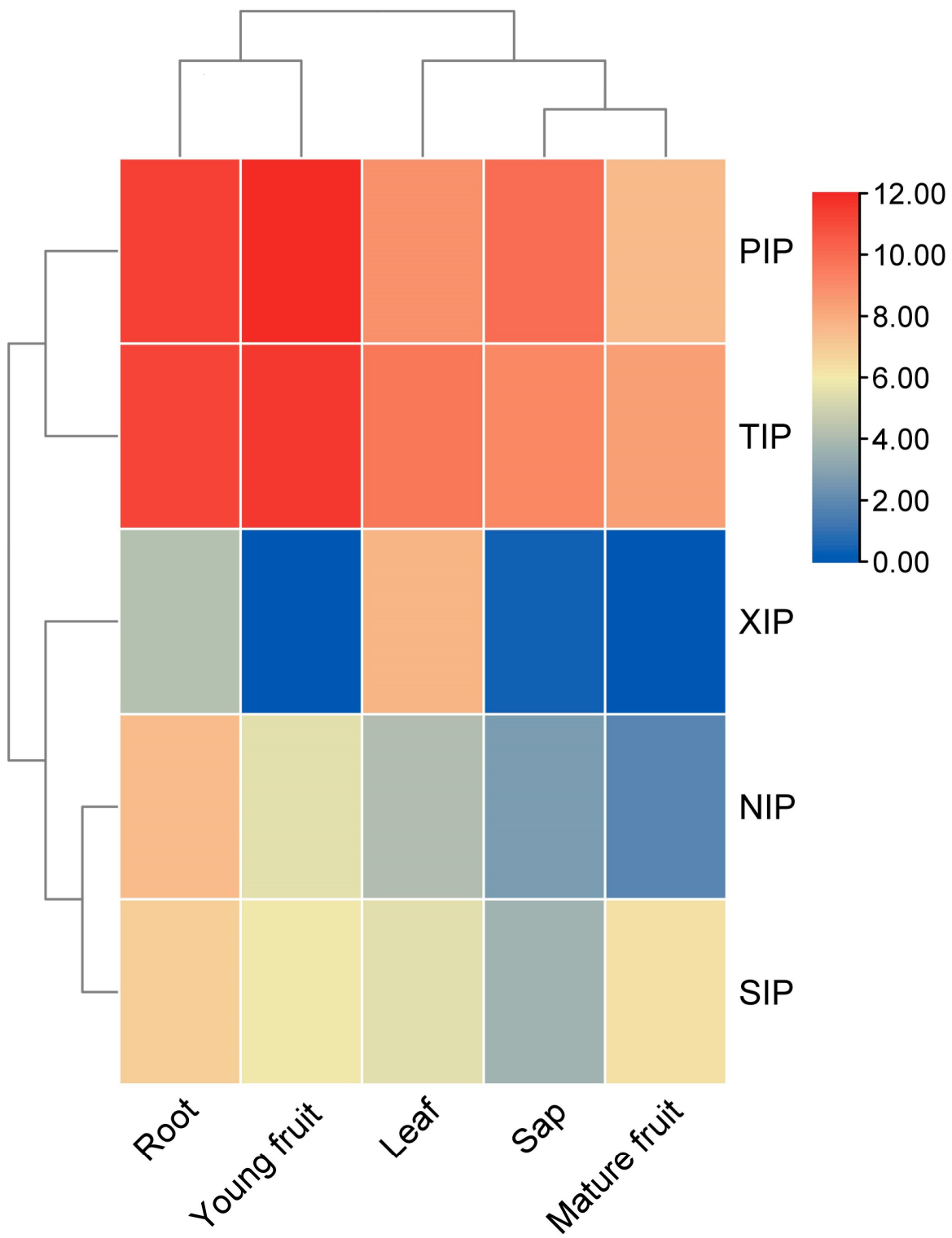

Supplement: Supplementary file 1 [file plants-12-03847-s001.zip › Figure S5.pdf]
